# ApoE Regulates the Development of Adult Newborn Hippocampal Neurons

**DOI:** 10.1523/ENEURO.0155-18.2018

**Published:** 2018-08-02

**Authors:** Yacine Tensaouti, Elizabeth P. Stephanz, Tzong-Shiue Yu, Steven G. Kernie

**Affiliations:** 1 Department of Pediatrics, Columbia University College of Physicians and Surgeons, New York, NY 10032; 2 Department of Neurology, Columbia University College of Physicians and Surgeons, New York, NY 10032

**Keywords:** ApoE, dentate gyrus, neurogenesis

## Abstract

Adult hippocampal neurogenesis occurs throughout life and is believed to participate in cognitive functions such as learning and memory. A number of genes that regulate adult hippocampal neurogenesis have been identified, although most of these have been implicated in progenitor proliferation and survival, but not in the development into fully differentiated neurons. Among these genes, apolipoprotein E (ApoE) is particularly compelling because the human ApoE isoform E4 is a risk factor for the development of Alzheimer’s disease, where hippocampal neurogenesis is reported to be dysfunctional. To investigate the effects of ApoE and its human isoforms on adult hippocampal neurogenesis and neuronal development, retroviruses carrying a GFP-expressing vector were injected into wild-type (WT), ApoE-deficient, and human targeted replacement (ApoE3 and ApoE4) mice to infect progenitors in the dentate gyrus and analyze the morphology of fully developed GFP-expressing neurons. Analysis of these adult-born neurons revealed significant decreases in the complexity of dendritic arborizations and spine density in ApoE-deficient mice compared with WT mice, as well as in ApoE4 mice compared with ApoE3. These findings demonstrate that ApoE deficiency and the ApoE4 human isoform both impair hippocampal neurogenesis and give insight into how ApoE may influence hippocampal-related neurological diseases.

## Significance Statement

Apolipoprotein E (ApoE) is known to regulate postnatal neurogenesis in the dentate gyrus of the hippocampus by directly affecting the proliferation of early progenitor cells. We found reduced complexity of adult-born granule cell dendritic arborizations as well as reduced spine density in ApoE-deficient and ApoE4 mice dentate gyrus neurons. These results provide strong evidence of impaired development of adult-born neurons in ApoE-deficient and ApoE4 mouse hippocampus, which may help to explain the higher risk of hippocampal-related neuropsychiatric diseases in humans carrying the E4 allele.

## Introduction

Increasing evidence suggests that some cognitive deficits, difficulty in learning new information and memory loss, are due to alterations in adult dentate gyrus neurogenesis, a key component of hippocampus-associated neurologic diseases such as major depressive disorder (Sahay and Hen, 2007), schizophrenia (Allen et al., 2016), Alzheimer's disease (AD; Perry et al., 2012; Hollands et al., 2016), traumatic brain injuries (Hong et al., 2016), and epilepsy (Cho et al., 2015). Therefore, understanding the mechanisms underlying adult neurogenesis is essential before hippocampal progenitor manipulation can be a viable target in treating or alleviating symptoms of neurologic and neurodegenerative diseases.

Despite increasing knowledge regarding the developmental steps that control hippocampal neurogenesis and the integration of adult-born granule neurons in the pre-existing circuitry, key regulatory genes underlying this process remain largely unknown. By comparing the expression profile of neural stem and progenitor cells (NSPCs) from mouse dentate gyrus at postnatal days 7 and 28, apolipoprotein E (ApoE) was identified as a potential regulator of adult neurogenesis (Gilley et al., 2011) and subsequently shown to regulate developmental and postnatal neurogenesis in the dentate gyrus by preventing overproliferation of NSPCs (Yang et al., 2011). In addition, ApoE deficiency is known to shift neural stem cell differentiation toward astrogenesis and away from neurogenesis (Li et al., 2009).

ApoE in the brain is produced and secreted primarily by astrocytes but is also expressed in type 1 neural stem cells (Hong et al., 2016). ApoE is involved in the regulation of lipid transport mostly from astrocytes to neurons, synaptogenesis, lipid intracellular homeostasis, β-amyloid clearance, and maintenance of the blood–brain barrier integrity (Vance et al., 2000; Mauch et al., 2001; Jiang et al., 2008; Bell et al., 2012). In humans, polymorphisms in ApoE result in the following three isoforms of the protein—ApoE2, ApoE3, and ApoE4. ApoE3 has been proposed as the “wild-type”(WT) allele because it is the most prevalent isoform in the population and is not associated with a disease phenotype (Hauser et al., 2011). The presence of ApoE4 in humans is the strongest known genetic risk factor associated with the development of late-onset Alzheimer’s disease as well as poorer outcomes after traumatic brain injury, with links to dementia and cognitive function deficits (Jordan et al., 1997; Teasdale et al., 1997; Merritt and Arnett, 2016). ApoE4 transgenic mice also exhibit impaired working memory in the radial arm maze (Hartman et al., 2001), and have been shown to exhibit abnormal neuronal maturation without a shift toward astrogenesis in the dentate gyrus (Li et al., 2009).

Given that several hippocampus-related neurologic diseases are influenced by both neurogenesis and ApoE genotype and that ApoE influences developmental and adult neurogenesis, we investigated the effect of ApoE human polymorphisms and ApoE deficiency on the activation of type 1 cells and their subsequent development into adult-born hippocampal neurons. To understand the myriad roles of ApoE in the brain, ApoE-deficient, human ApoE2, ApoE3, and ApoE4 targeted replacement mice have been widely used for a variety of investigations. Although it is known that ApoE influences hippocampal neurogenesis by acting as a negative regulator of proliferation in NSPCs, it remains unknown how it might affect ongoing development of adult-born neurons. Given that ApoE is expressed predominantly by astrocytes, we hypothesized that the adult newborn neuron development of dendrites and spines might be regulated by ApoE, where its expression in astrocytes is most abundant. Here, we demonstrate that not only does ApoE alter the proliferation of NSPCs, but it also directs changes in the complexity of mature newborn neurons at the dendritic and spine level that appear to be mediated by the close approximation of astrocytes and developing dendrites.

## Materials and Methods

### Animals

Experimental animals were humanely housed and cared for under the supervision of the Institute of Comparative Medicine. C57BL/6J mice (used as the wild type for all experiments) as well as ApoE-deficient mice were purchased from The Jackson Laboratory. Purchased ApoE-deficient mice were crossed with and maintained under C57BL/6J genetic background as described previously (Yang et al., 2011). ApoE3 and ApoE4 targeted replacement mice were obtained from Taconic (Table 1). All experimental procedures were in accordance with the National Institutes of Health guidelines and were approved by the Institutional Animal Care and Use Committee at Columbia University College of Physicians and Surgeons.

**Table 1.** Key resources table, highlighting the genetically modified organisms and strains, viruses and software essential to reproduce results presented in the manuscript

Reagent or resource	Source	Identifier	
Bacterial and virus strains			
Moloney murine leukemia viral vectors, RV-CAG-eGFP or RV-CAG-eGFPcre	GT3 Core Facility of the Salk Institute	NIH-NCI CCSG: P30 014195, NINDS R24 Core Grant, NEI	
Experimental models: organisms/strains			
B6.129P2-Apoetm1Unc/J	The Jackson Laboratory	Catalog #JAX:002052; RRID:IMSR_JAX:002052, https://www.jax.org/strain/002052	
B6.129P2-Apoetm2(APOE*3)Mae N8	Taconic	Catalog #TAC:1548; RRID:IMSR_TAC:1548, https://www.taconic.com/transgenic-mouse-model/apoe3	
B6.129P2-Apoetm3(APOE*4)Mae N8	Taconic	Catalog #TAC:1549; RRID:IMSR_TAC:1549, https://www.taconic.com/transgenic-mouse-model/apoe4	
B6;129S6-Gt(ROSA)26Sortm9(CAG-tdTomato)Hze/J	The Jackson Laboratory	Catalog #JAX: 007909; RRID:IMSR_JAX:007905, https://www.jax.org/strain/007905	
C57BL/6J	The Jackson Laboratory	Catalog #JAX:000664; RRID:IMSR_JAX:000664, https://www.jax.org/strain/000664	
Software			
Adobe Photoshop	Adobe		RRID:SCR_014199, https://www.adobe.com/products/photoshop.html
AutoQuant	Media Cybernetics		RRID:SCR_002465, http://www.mediacy.com/index.aspx?page=AutoQuant
Prism	GraphPad Software		RRID:SCR_015807, https://www.graphpad.com/scientific-software/prism/
Neurolucida	mbf BIOSCIENCE		RRID:SCR_001775, http://www.mbfbioscience.com/neurolucida
Stereo Investigator	mbf BIOSCIENCE		RRID:SCR_002526, http://www.mbfbioscience.com/stereo-investigator

RRID, Research resource identifiers (https://scicrunch.org/resources).

### Retroviral injections

To highlight the potential physical interaction between ApoE-expressing astrocytes and adult-born granule cells in the dentate gyrus, one 6-week-old tdTomato reporter mouse (Table 1) was injected with an eGFPcre-expressing retrovirus [1 × 10^9^ transducing units (TU)/ml] into the dentate gyrus [Moloney Murine Leukemia Viral vectors were generated by the GT3 Core Facility of the Salk Institute (La Jolla, CA) with funding by National Institutes of Health (NIH)-National Cancer Institute (NCI) Cancer Center Support Grant (CCSG) P30-014195, a National Institute of Neurological Disorders and Stroke (NINDS) R24 Core Grant; and funding from National Eye Institute (NEI)] inducing robust expression of tdTomato fluorescence in infected cells 4 weeks postinjection. Twenty-one 6-week-old mixed-sex mice were stereotactically injected with an enhanced green fluorescent protein (eGFP)-expressing retrovirus to infect adult neural progenitors in the dentate gyrus (Moloney Murine Leukemia Viral vectors were generated by the GT3 Core Facility of the Salk Institute with funding from NIH-NCI CCSG P30-014195, an NINDS R24 Core Grant and funding from NEI). The constitutive expression of eGFP in the infected cells allowed for the tracing of infected NSPCs. One microliter of packaged GFP-expressing retrovirus (1 × 10^9^ TU/ml) was infused, using a microinfusion pump (KD Scientific) linked to a 10 μl syringe (model #801, Hamilton), at the rate of 0.1 μl/min into the dentate gyrus with the following coordinates: antero/posterior, −2.0/−2.5 mm; medio/lateral, ±1.55/±2 mm; dorso/ventral, −2.0/−2.25 mm.

### Tissue processing and immunohistochemistry

Four weeks after retroviral injections, the time it takes for a newborn granular neuron to be mature and become integrated into the pre-existing hippocampal circuitry (Toni and Schinder, 2015), animals were deeply anesthetized with isoflurane through the whole period of perfusion. Transcardiac perfusion was performed with 50 ml of 1× PBS, followed by 100 ml of 4% paraformaldehyde (PFA) in 1× PBS. Whole brains were then dissected and immersed in 4% PFA/1× PBS overnight for postfixation. Then, brains were embedded in 3% agarose/1× PBS, and serial 50 μm sections were cut with a vibratome (model VT1000S, Leica). All sections encompassing the hippocampus were collected sequentially in six-well plates. Free-floating sections were used for immunohistochemistry. All brain sections containing the hippocampus also had GFP-expressing adult-born granules cells, showing the efficacy of injections. All samples were kept at −20°C in antifreeze solution (30% glycerol/30% ethylene glycol in PBS).

A set of sections containing every sixth slice was washed with PBS 1× to remove antifreeze solution (3× 5 min) then samples were permeabilized with 0.3% Triton X-100/1× PBS (PBST; 3× 10 min) at room temperature (RT). Samples were then blocked for 1 h at RT with PBST containing 5% normal goat serum (NGS) or normal donkey serum (NDS; Jackson ImmunoResearch Laboratories). Samples were then incubated with primary antibodies (Table 2, references and concentrations used) in PBST with 5% NGS or NDS at 4°C overnight or at RT [with 0.02% (w/v) sodium azide] overnight. The following day, sections were washed with PBST three times and incubated with secondary antibodies (Alexa Fluor 488, Alexa Fluor 594, Alexa Fluor 647, or biotin-conjugated antibodies, Jackson ImmunoResearch Laboratories) for 3 h at RT. Sections were then washed three times with PBS and incubated with Alexa Fluor 488-, Alexa Fluor 594-, or Alexa Fluor 647-conjugated streptavidin antibodies (Jackson ImmunoResearch Laboratories) for 2 h at RT if biotin-conjugated antibodies were used. Sections were finally rinsed three times with PBS, then mounted on slides with Vectashield Mounting Medium with DAPI (catalog #H-1500, Vector Laboratories) and sealed with coverslips. For BrdU staining, sections were denatured with 1N HCl for 45 min in a 37°C water bath before normal staining procedure.

**Table 2.** List of antibodies used and concentrations

Target	Description	Provider	Dilution	RRID
GFP	Rabbit polyclonal	Invitrogen	1:500	Catalog #A-11122; RRID:AB_221569
BrdU	Rat monoclonal	Abcam	1:500	Catalog #ab6326; RRID:AB_305426
GFAP	Guinea Pig polyclonal	MyBioSource	1:500	Catalog #MBS834682
Ki67	Rabbit monoclonal	Thermo Fisher Scientific	1:500	Catalog #RM-9106-S0; RRID:AB_2341197
Prox1	Rabbit polyclonal	Abcam	1:500	Catalog #ab101851; RRID:AB_10712211
ApoE	Goat polyclonal	EMD Millipore	1:5000	Catalog #AB947; RRID:AB_2258475
Rabbit	Biotin Goat polyclonal	Jackson ImmunoResearch Laboratories	1:200	Catalog #111-065-003; RRID:AB_2337959
Goat	Biotin Donkey polyclonal	Jackson ImmunoResearch Laboratories	1:200	Catalog #705-065-147; RRID:AB_2340397
Rat	A488 Donkey polyclonal	Jackson ImmunoResearch Laboratories	1:200	Catalog #712-546-153; RRID:AB_2340686
Rabbit	A488 Donkey polyclonal	Jackson ImmunoResearch Laboratories	1:200	Catalog #711-545-152; RRID:AB_2313584
Guinea pig	A647 Donkey polyclonal	Jackson ImmunoResearch Laboratories	1:200	Catalog #706-605-148; RRID:AB_2340476
Rabbit	A647 Donkey polyclonal	Jackson ImmunoResearch Laboratories	1:200	Catalog #711-605-152; RRID:AB_2492288
Biotin	A488 Streptavidin polyclonal	Jackson ImmunoResearch Laboratories	1:200	Catalog #016-540-084; RRID:AB_2337249
Goat serum	Normal Goat Serum antibody	Jackson ImmunoResearch Laboratories	5%	Catalog #005-000-121; RRID:AB_2336990
Donkey serum	Normal Donkey Serum antibody	Jackson ImmunoResearch Laboratories	5%	Catalog #017-000-121; RRID:AB_2337258

RRID, Research resource identifier (https://scicrunch.org/resources).

### BrdU and Prox1 quantification

BrdU was administered via intraperitoneal injection (100 mg/kg) for 3 consecutive days to 19 6-week old wild-type C56JBL/6, ApoE-deficient, ApoE3 and ApoE4 mice from males and females, and brains were harvested for further analyses at 10 weeks of age after transcardiac perfusion as described above. To determine the number of BrdU-positive and Brdu-Prox1 double-positive cells in both the subgranular zone (SGZ) and granular cell layer (GCL) of the dentate gyrus, an unbiased stereological approach was used. Samples were analyzed using a Zeiss microscope (Axio Imager M2, Zeiss) with a Hamamatsu camera (Orca-R2, Hamamatsu). The cells were counted using an optical fractionator and stereological image analysis software (RRID:SCR_002526). SGZ and GCL of the dentate gyrus were traced under a 10× objective lens. The stereological software randomly selected sample grids (100 × 100 μm), and cells within the counting frames (50 × 50 μm) were counted under a 40× objective. To avoid artifacts that resulted from sectioning, a dissector height of 30 μm was used. In addition to counting BrdU-positive cells, colocalization of BrdU and Prox1 was also quantified. The software estimated cell number by using weighted section thickness to yield an absolute number of cells in the hippocampus. Following each count, the software also calculated a coefficient of error. To ensure that the estimated counts were accurate, the coefficient of error for each included count was <0.1.

### Nestin, Ki-67, and GFAP quantification

To determine the number of nestin-positive, Ki67-positive, nestin-GFAP double-positive, and nestin-GFAP-Ki67 triple-positive cells in the SGZ of the dentate gyrus, 10-week-old wild-type C56JBL/6, ApoE-deficient, ApoE3, and ApoE4 mice samples (4 mice/group) were sectioned, stained, and analyzed using stereological quantification as described. The SGZ of the dentate gyrus was traced under a 20× objective lens. The stereological software randomly selected sample grids (200 × 200 μm), and cells within the counting frames (100 × 100 μm) were counted under a 63× oil-objective. To avoid artifacts that resulted from sectioning, a dissector height of 30 μm was used. The software estimated cell numbers by using weighted section thickness to yield an absolute number of cells in the hippocampus. To ensure the estimated counts were accurate, the coefficient of error for each included count was <0.1. Representative images were acquired using a Laser Scan Confocal Microscope (TCS SP8, Leica), and images were deconvolved using Autoquant (RRID:SCR_002465).

### Analysis of dendritic morphology and spine density

Immunostained sections were visualized using a Zeiss microscope (Axio Imager M2, Zeiss) with a Hamamatsu camera (Orca-R2, Hamamatsu). Stack images (1 μm interval on *z*-axis) were acquired using an optical fractionator and stereological image analysis software (RRID:SCR_002526) under a 20× objective. A minimum of 10 neurons/mouse underwent three-dimensional (3D) reconstruction using the Neurolucida360 software (RRID:SCR_001775; Dickstein et al., 2016) to analyze the morphology of those cells: measurement of the length of the proximal branch before the first division (first branch), the cumulative length of the dendritic tree, the number of nodes, and the span of the dendritic tree, and a Sholl analysis were performed.

Sholl analysis (see Figs. 3*G*, 5*G*) revealed that the number of dendrites of adult-born granule cells peaks at ∼100 μm from the soma, and we found statistical differences among the different genotypes. For further investigation, spine analysis was performed on dendrites in the molecular layer (ML) only. Image stacks were obtained using a Laser Scan Confocal Microscope (TCS SP8, Leica), under a 63× oil-objective lens, with 5× digital zoom, intervals of 0.1 μm along the *z*-axis, leading to a pixel size of 57.21 nm (numerical aperture = 1.44; resolution = 512 × 512; frame average = 4). Images were deconvolved using Autoquant (RRID:SCR_002465), 10 μm dendritic fragments were randomly picked for tracing (one fragment/dendrite) and were analyzed by an experimenter blindly using the automated software Neurolucida 360 (RRID:SCR_001775), which performed 3D analysis of dendritic length and spine number (Dickstein et al., 2016). After automated detection of spines, a manual examination was performed (i.e., adding, deleting, merging, or splitting identified objects). Because of spherical aberrations, objects on the *z*-axis appeared blurred and smeared, potentially leading to an underestimation of the spine density, added to the fact that the diameters of dendrites vary too, we elected to express the number of spines as a function of the fragment length instead of using its surface, which could potentially lead to confounding results (Dumitriu et al., 2011).

### Statistical analysis

All statistical analyses were performed using GraphPad Prism (RRID:SCR_015807). The normality of data were assessed using the Shapiro–Wilk test, and variance was assessed using Levene’s test. Results are presented as the mean ± SEM. Statistical details are presented in text and summarized in Table 3. The unpaired Student’s *t* test was used to test for differences between wild-type and ApoE-deficient animals, and between ApoE3 and ApoE4 animals since these two groups [ApoE knock-out (KO)/wild-type and ApoE3/ApoE4 were on distinct genetic backgrounds]. Two-way ANOVA with Fisher’s least significant difference (LSD) *post hoc* test was used to test differences in Sholl analysis. The effect size was calculated using Cohen’s *d* statistic. A value of *p* < 0.05 was considered statistically significant.

**Table 3.** Summary of statistics.

	Table analyzed	Statistical test	*p* value	Size effect	*t* value	df	*R* ^2^	*F* value
WT vs KO	BrdU counts	Unpaired Student’s *t* test	0.8469	0.60	0.494	8	0.029	9.72 *p* = 0.049
	BrdU-Prox1 colocalization	Unpaired Student’s *t* test	0.6784	0.23	0.3652	8	0.016	5.473 *p* = 0.1284
	% Newborn neurons	Unpaired Student’s *t* test	0.2214	0.19	0.2983	8	0.011	1.266 *p* = 0.8249
	Nestin-Ki67-GFAP	Unpaired Student’s *t* test	0.0376	1.88	2.658	6	0.5408	8.054 *p* = 0.1204
	Nestin-GFAP	Unpaired Student’s *t* test	.0291	2.02	2.851	6	0.575	1.639 *p* = 0.6947
	Ki67	Unpaired Student’s *t* test	0.0333	2.47	2.75	6	0.5576	1.015 *p* = 0.9903
	Length of the first branch	Unpaired Student’s *t* test	0.1658	0.23	1.393	149	0.0128	1.284 *p* = 0.2817
	Number of nodes	Unpaired Student’s *t* test	0.0001	0.65	3.939	149	0.0943	1.617 *p* = 0.0406
	DA cumulative length	Unpaired Student’s *t* test	0.1117	0.26	1.6	149	0.0169	1.133 *p* = 0.5929
	DA span	Unpaired Student’s *t* test	0.0032	0.49	2.967	149	0.05578	3.387 *p* = 0.0001
	Sholl analysis							
	Intersection effect	Two-way ANOVA	<0.0001					*F*_(26,4023)_ = 2.488
	Row effect	Two-way ANOVA	<0.0001					*F*_(26,4023)_ = 163.7
	Column effect	Two-way ANOVA	<0.0001					*F*_(1,4023)_ = 37.46
	Spine density	Unpaired Student’s *t* test	<0.0001	1.13	4.163	107	0.1394	1.016 *p* = 0.9479
E3 vs E4	BrdU Counts	Unpaired Student’s *t* test	0.8961	0.094	0.135	7	0.002612	2.668 *p* = 0.4463
	BrdU-Prox1 colocalization	Unpaired Student’s *t* test	0.5838	0.40	0.574	7	0.04498	3.759 *p* = 0.3053
	% Newborn neurons	Unpaired Student’s *t* test	0.1097	1.22	1.832	7	0.324	1.234 *p* = 0.8143
	Nestin-Ki67-GFAP	Unpaired Student’s *t* test	0.486	0.52	0.7419	6	0.6185	1.875 *p* = 0.6185
	Nestin-GFAP	Unpaired Student’s *t* test	0.3700	0.69	0.9689	6	0.1351	1.191 *p* = 0.8891
	Ki67	Unpaired Student’s *t* test	0.2795	0.84	1.189	6	0.1906	2.6 *p* = 0.4533
	Length of the first branch	Unpaired Student’s *t* test	0.8949	0.023	0.132	142	0.000123	1.09 *p* = 0.7196
	Number of nodes	Unpaired Student’s *t* test	0.0007	0.59	3.472	142	0.07826	1.408, *p* = 0.1542
	DA cumulative length	Unpaired Student’s *t* test	<0.0001	0.7	4.127	142	0.1071	1.783, *p* = 0.0166
	DA Span	Unpaired Student’s *t* test	0.5978	0.09	0.528	142	0.001965	1.296, *p* = 0.2749
	Sholl analysis							
	Intersection effect	Two-way ANOVA	0.0013					*F*_(26,3834)_ = 2.048
	Row effect	Two-way ANOVA	<0.0001					*F*_(26,3834)_ = 144.7
	Column effect	Two-way ANOVA	<0.0001					*F*_(1,3834)_ = 112.8
	Spine density	Unpaired Student’s *t* test	<0.0001	1.13	5.278	88	0.2405	2.08 *p* = 0.0173

D, Dendritic arborization. Cohen’s *d* value was calculated as $d=\frac{Mean1-Mean2}{SDpooled}$ with $SDpooled=\sqrt{\frac{{SD}_{1}^{2}+{SD}_{2}^{2}}{2}}$ , *d* = 0.2, *d* = 0.5, and *d* = 0.8 corresponding to small, medium, and large size effects, respectively

## Results

### ApoE expression during dentate gyrus neuronal maturation

**Figure 1.**
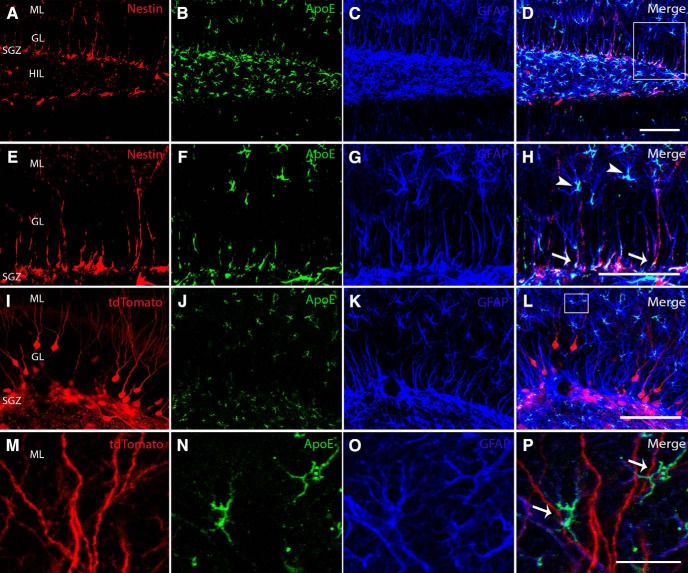
Type 1 NSPCs express nestin, ApoE, and GFAP, while mature granule cells make connections with astrocytes expressing ApoE and GFAP. ***A–D***, Representative confocal images of the dentate gyrus illustrate nestin-expressing (red), ApoE-expressing (green), and GFAP-expressing (blue) cells, along with a merged image of all channels. ***E–H***, High-power representative confocal images taken from the region in the inset in ***D*** illustrate nestin-expressing (red), ApoE-expressing (green), and GFAP-expressing (blue) cells, along with a merged image of all channels. White arrows indicate nestin-ApoE-GFAP triple-positive cells, representing type 1 NSPCs. ***I–L***, Representative confocal images of the dentate gyrus illustrate tdTomato-expressing (red), ApoE-expressing (green), and GFAP-expressing (blue) cells, along with a merged image of all channels. ***M–P***, High-power representative confocal images taken from the region in the inset in ***L*** illustrate tdTomato-expressing (red), ApoE-expressing (green), and GFAP-expressing (blue) cells, along with a merged image of all channels. White arrows indicate GFAP and ApoE coexpressing astrocytes. GL, Granule layer; HIL, hilus. Scale bars: ***D***, 100 µm; ***H***, 75 µm; ***L***, 100 µm; ***P***, 25 µm.

ApoE demonstrates widespread expression in the dentate gyrus, both in nestin-expressing neural stem and progenitor cells in the subgranular zone as well as GFAP-expressing astrocytes in the hilus and molecular layer (Fig. 1*A–H*). To determine the expression of ApoE in approximation to the dendritic arborizations of adult-born neurons, a retrovirus expressing Cre was injected stereotactically into the dentate gyrus subgranular zone of ROSA-26 tdTomato reporter mice to infect dividing NSPCs, which then express tdTomato. Four weeks after injection, mice were sectioned and stained for ApoE and GFAP to demonstrate astrocytic expression of ApoE. We observed that ApoE-expressing astrocytes are in very close proximity and appear to be physically interacting with the dendrites of just maturing adult-born neurons (Fig. 1*I–P*).

### Type 1 neural stem and progenitor proliferation in wild-type and ApoE-deficient mice

**Figure 2.**
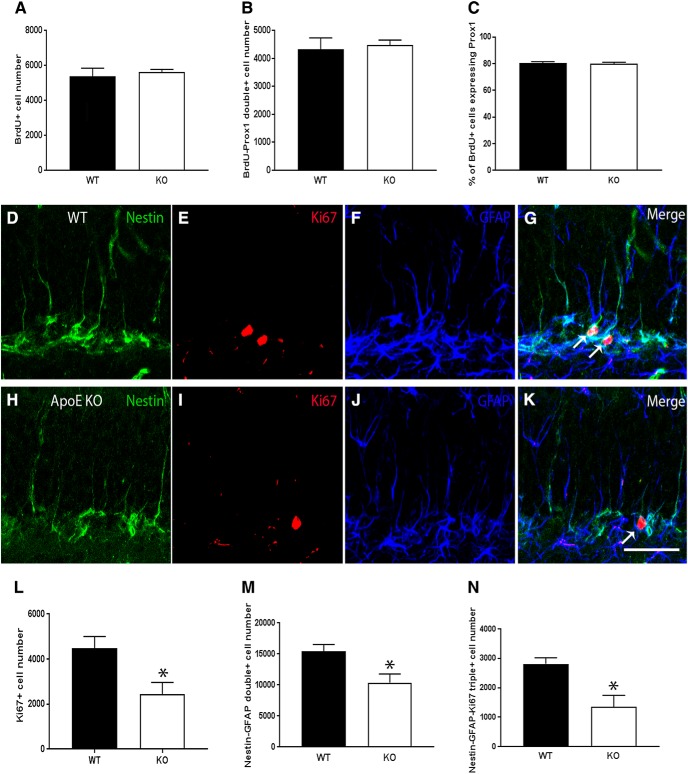
ApoE deficiency leads to a decrease in overall proliferation, the number of type 1 NSPCs, and type 1 NSPC proliferation in the dentate gyrus, while leaving the ability of newborn cells to survive and differentiate into granular neurons unimpaired. ***A–C***, Unbiased stereological quantification of the number of BrdU-positive cells (***A***), the number of BrdU-Prox1 double-positive cells (***B***), and the percentage of BrdU-positive cells that also express Prox1 (***C***). ***D–K***, Representative confocal images of the SGZ in WT and ApoE-deficient mice illustrate nestin-expressing (green), Ki67-expressing (red), and GFAP-expressing (blue) cells, along with a merged image of all channels. White arrows indicate nestin-Ki67-GFAP triple-positive cells, representing actively proliferating type 1 NSPCs. ***L–N***, Unbiased stereological quantification of the number of Ki67-positive cells (***L***), the number of nestin-GFAP double-positive cells (***M***), and the number of nestin-GFAP-Ki67 triple-positive cells (***N***). Results are expressed as the mean ± SEM. Unpaired Student’s *t* tests, *p < 0.05. Scale bar, ***K***, 50 µm.

To investigate the effect of ApoE on neuronal survival and differentiation in the hippocampus, BrdU (100 mg/kg) was administered via intraperitoneal injection for three consecutive days to 6-week-old wild-type and ApoE-deficient mice, and brains were harvested for further analysis 4 weeks later, the time it takes for immature neurons to express mature neuronal markers. Using unbiased stereology, BrdU-positive cells, and BrdU-Prox1 double-positive cells, representing dividing cells that became granular neurons, were quantified; and total counts were estimated. Additionally, to determine what percentage of newborn hippocampal cells became mature granular neurons, the number of BrdU-Prox1 colocalized cells was divided by the total number of BrdU-positive cells and multiplied by 100. No significant differences were found between wild-type and ApoE-deficient mice for total BrdU-positive cells (Fig. 2*A*; WT, 5368 ± 474.2; KO, 5614 ± 152.1; unpaired Student’s *t* test), BrdU-Prox1 colocalization (Fig. 2*B*; WT, 4320 ± 415.5; KO, 4485 ± 177.6; unpaired Student’s *t* test), or for the percentage of newborn neurons (Fig. 2*C*; WT, 80.30 ± 1.136; KO, 79.79 ± 1.278; unpaired Student’s *t* test), indicating that ApoE deficiency does not impair the ability of newborn hippocampal cells to survive and differentiate into granular neurons 4 weeks after BrdU administration.

Because there was no significant change in the ability of newborn hippocampal cells to survive and differentiate into neurons in the absence of ApoE, and because previous studies have determined that ApoE expression is high in type 1 NSPCs (Hong et al., 2016), the proliferation of NSPCs was next analyzed to determine whether ApoE deficiency affects type 1 NSPC activation specifically. Four samples from each genotype were sectioned and immunostained for nestin, Ki67, and GFAP, and were quantified using unbiased stereology. Representative images of the SGZ are shown for wild-type and ApoE-deficient mice (Fig. 2*D–K*). Compared with wild-type controls, ApoE-deficient brains exhibited significantly reduced Ki67 staining (Fig. 2*L*; WT, 4477 ± 524.0; KO, 2433 ± 528.0; unpaired Student’s *t* test: *p* = 0.033, *d* = 2.47, *t* = 2.75), indicating a reduction in overall proliferation at 10 weeks of age. Additionally, in the absence of ApoE, there were significantly fewer nestin-GFAP colocalized cells in the dentate gyrus (Fig. 2*M*; WT, 15430 ± 1088.0; KO, 10390 ± 1393.0; unpaired Student’s *t* test: *p* = 0.0291, *d* = 2.02, *t* = 2.85), representing a reduction in the total number of NSPCs. Further, ApoE-deficient samples had significantly fewer nestin-GFAP-Ki67 triple-positive cells (Fig. 2*N*; WT, 3592 ± 295.0; KO, 1351 ± 280; unpaired Student’s *t* test: *p* = 0.0376, *d* = 1.88, *t* = 2.66), a decrease suggesting that ApoE deficiency specifically affects NSPC proliferation.

### ApoE deficiency impairs the dendritic complexity of adult-born dentate gyrus neurons

After proliferating in the SGZ of the dentate gyrus, NSPCs migrate short distances to the inner segment of the GCL and start to expand and develop their dendritic trees into the ML (Kempermann et al., 2015). To assess the effect of ApoE on the maturation of adult-born granule cell dendritic trees in the dentate gyrus, 6-week-old C56JBL/6 mice (*n* = 4 mice, 78 neurons) and ApoE-deficient mice (*n* = 7 mice, 73 neurons) were stereotactically injected with a GFP-expressing retrovirus and killed 4 weeks after injection to make 3D reconstructions. The morphology of these neurons was analyzed based on the length of the proximal branch before the first division (the first branch), the cumulative length of the dendritic tree, the number of nodes (every time a branch divides), the span of the dendritic tree, and Sholl analysis.

**Figure 3.**
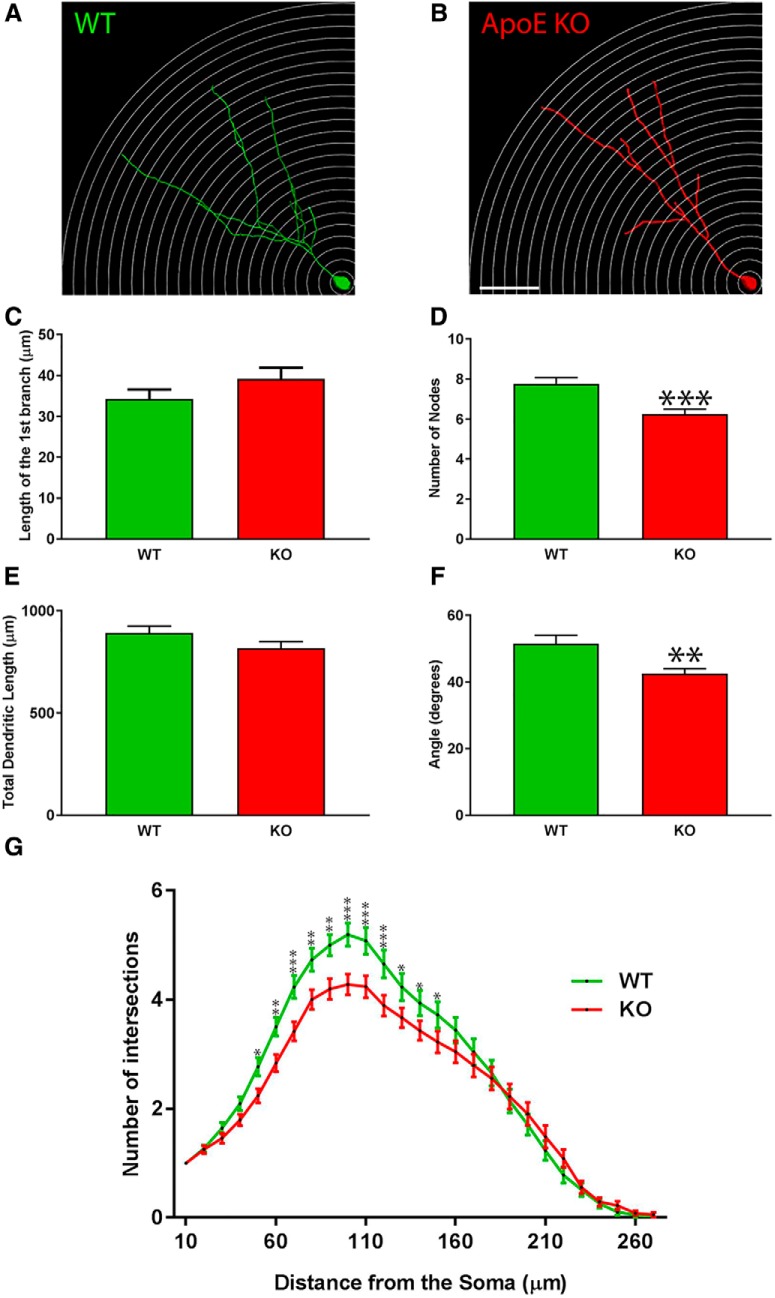
The complexity as well as the total dendritic length of ApoE-deficient adult-born granule cells are significantly reduced. ***A***, ***B***, Representative 3D-reconstructed adult-born granule cells (4 weeks after stereotactic injections), one circle every 10 µm. ***C***, Distance between the soma and the first dendritic division (in µm). ***D***, Nodes were defined as the number of divisions of dendritic branches. ***E***, Cumulative length of dendritic arborization (in µm). ***F***, Span of the dendritic arborization when projected on two dimensions. ***G***, Sholl analysis: the number of dendritic intersections as a function of the distance from the soma: wild type, 78 neurons/4 mice; ApoE-deficient, 73 neurons/7 mice. Results are expressed as the mean ± SEM. Independent *t* test and two-way-ANOVA with uncorrected Fisher’s LSD *post hoc* tests were used: *p < 0.05, **p < 0.01, ***p < 0.001. Scale bar, ***B***, 50 µm.

Representative pictures of 3D-reconstructed adult-born granule cells are shown for both WT and ApoE-deficient mice (Fig. 3*A*,*B*). We did not observe any difference in the location of newborn granule cells in the dentate gyrus of WT and ApoE-deficient mice, because, apart from rare exceptions, all adult-born granule cells were found in the inner one-third of the GCL. No significant differences were found in the length of the first branch between WT and ApoE-deficient mice (Fig. 3*C*; WT, 36.41 ± 1.97 µm; KO, 37.59 ± 2.19 µm; unpaired Student’s *t* test). After the first dendritic division, new dendrites continue to grow in the GCL and ML, sensing the environment and bifurcating several times (Kempermann et al., 2015). We found a significant decrease in the number of nodes in ApoE-deficient mice (6.25 ± 0.24 µm) compared with WT mice (7.77 ± 0.29 µm; Fig. 3*D*; unpaired Student’s *t* test: *p* = 0.001, *d* = 0.65, *t* = 3.94). However, no significant differences have been found in the total dendritic length (Fig. 2*E*; WT, 875.2 ± 26.01 µm; KO, 785.5 ± 25.76 µm; unpaired Student’s *t* test). We also found that the dendritic tree span (42.54 ± 1.45°) of ApoE-deficient mice was significantly reduced compared with that of WT mice (51.47 ± 2.58°; Fig. 3*F*; unpaired Student’s *t* test: *p* = 0.0035, *d* = 0.49, *t* = 2.97).

We then analyzed the distribution of adult-born granule cell dendrites in the dentate gyrus by performing Sholl analysis (Fig. 3*A*,*B*, representative pictures). Two-way ANOVA revealed a highly significant effect of the genotype and the distance from the soma on the number of intersections (*p* < 0.0001, *F*_(1,4023)_ = 37.46 and *p* < 0.0001, *F*_(26,4023)_ = 163.7, respectively), as well as a highly significant interaction between those two factors (*p* < 0.0001, *F*_(26,4023)_ = 2.488). *Post hoc* analysis did not demonstrate a change in the number of proximal dendrites (from 10 to 50 µm) among the different genotypes (Fig. 3*G*; uncorrected Fisher’s LSD *post hoc* tests) consistent with the fact that we did not find any difference in the occurrence of the first dendritic division (Fig 3*C*). However, we found that ApoE-deficient adult-born neurons had significantly fewer dendritic branches from 50 to 100 µm from the soma when compared to WT mice. For both groups, the dendrites reached the ML and the number of dendrites peaked at 100 µm. Furthermore, ApoE-deficient granule cells demonstrated significantly fewer interactions on Sholl analysis than WT cell at 100 to 150 µm from the soma (Fig. 3*G*; uncorrected Fisher’s LSD *post hoc* tests). No differences were observed 150 µm away from the soma.

### Hippocampal neuronal maturation in ApoE3 and ApoE4 mice

While there were no significant differences between ApoE-deficient and wild-type samples in the ability of newborn hippocampal cells to survive and differentiate into granular neurons, we next used unbiased stereological quantification to determine whether the presence of either the human ApoE3 or ApoE4 isoform affects these processes (*n* = 5 mice for ApoE3; *n* = 4 mice for ApoE4). Similar to the wild-type and ApoE-deficient mice, no significant differences were found between ApoE3 and ApoE4 mice for total BrdU-positive cells (E3, 4032 ± 368.3; E4, 3968 ± 252.1; unpaired Student’s *t* test, results not shown), BrdU-Prox1 colocalization (E3, 3216 ± 290.3; E4, 3009 ± 167.4; unpaired Student’s *t* test) or for the percentage of newborn neurons (E3, 79.79 ± 1.322; E4, 75.98 ± 1.642, unpaired Student’s *t* test), suggesting that the presence of either human isoform does not differentially impair the ability of newborn hippocampal cells to survive and differentiate into granular neurons 4 weeks after BrdU administration.

As there were no significant differences in neuronal survival and maturation between ApoE3 and ApoE4 mice, levels of type 1 NSPC proliferation were next studied to determine whether the human isoforms had differing effects on type 1 NSPC activation specifically. Four samples from each genotype were sectioned and immunostained for nestin, Ki67, and GFAP, and analyzed using unbiased stereological quantification. Unlike what we observed in wild-type and ApoE-deficient mice, there was no significant difference in Ki67 counts between ApoE3 and ApoE4 mice (E3, 2969 ± 224.8; E4, 3476 ± 362.5; unpaired Student's *t* test), indicating that the human isoforms do not differentially affect overall proliferation at 10 weeks of age. In addition, there was no significant difference in nestin-GFAP colocalization (E3, 11740 ± 1410.0; E4, 9887 ± 1292.1; unpaired Student's *t* test) indicating similar numbers of type 1 NSPCs in each genotype. Finally, there was no significant difference in the number of nestin-GFAP-Ki67 triple-positive cells between genotypes (E3, 1744 ± 187.6; E4, 1980 ± 256.9; unpaired Student’s *t* test), suggesting that the human isoforms lead to similar levels of NSPC proliferation.

### Adult-born granule neurons in ApoE4 mice demonstrate less complexity than that seen in ApoE3

**Figure 4.**
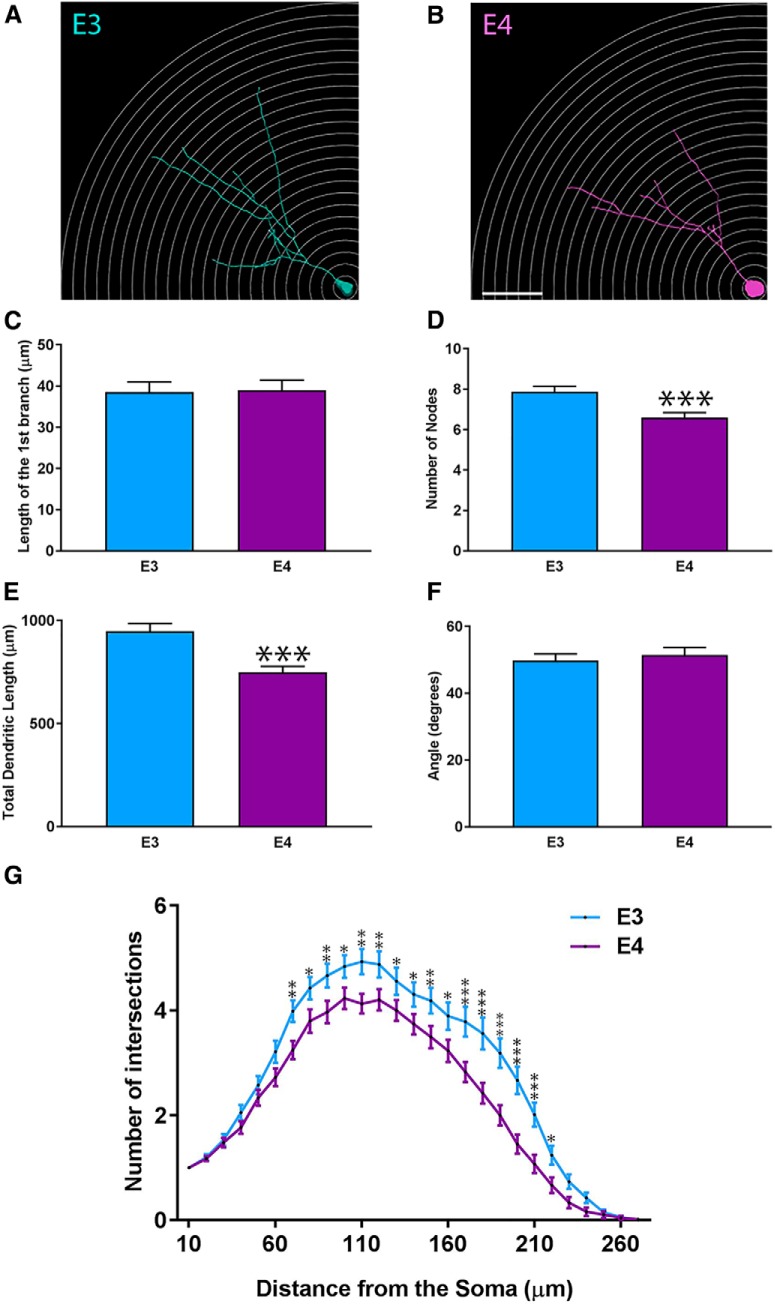
The complexity and the total dendritic length of ApoE4 adult-born granule cells are significantly reduced. ***A***, ***B***, Representative 3D-reconstructed adult-born granule cells (4 weeks after stereotactic injections), one circle every 10 µm. Representative picture of 4-week-old retrovirally labeled adult-born granule cells in the dentate gyrus. ***C***, Distance between the soma and the first dendritic intersection (in µm). ***D***, Nodes defined as the number of divisions of dendritic branches. ***E***, Cumulative length of dendritic arborizations (in µm). ***F***, Span of the dendritic arborizations when projected on two dimensions. ***G***, Sholl analysis: number of dendritic intersections as a function of the distance from the soma. ApoE3, 75 neurons/6 mice; ApoE4, 69 neurons/4 mice. Results are expressed as the mean ± SEM. Independent *t* test and two-way ANOVA with uncorrected Fisher’s LSD *post hoc* tests were used: *p < 0.05, **p < 0.01, ***p < 0.001, ****p < 0.0001. Scale bar, ***B***, 50 µm.

After observing an effect of ApoE deficiency on the dendritic morphology of adult-born granule cells in the dentate gyrus (i.e., reduced dendritic complexity, span, and number of branches in both GCL and ML), we next investigated the effect of human ApoE3 and ApoE4 on the development of mouse dentate gyrus adult born granule cell dendritic trees. Representative 3D-reconstructed adult-born granule cells are shown (Fig. 4*A*,*B*) for both ApoE3 mice (*n* = 6 mice, 75 neurons) and ApoE4 mice (*n* = 4, 69 neurons). No significant differences were found in the length of the first branch (Fig. 4*C*; E3, 38.99 ± 2.14 µm; E4, 39.57 ± 2.11 µm; unpaired Student’s *t* test). However, a significant decrease in the number of nodes was observed in ApoE4 mice (6.37 ± 0.19 µm) compared with ApoE3 mice (7.59 ± 0.22 µm; Fig. 4*D*; unpaired Student’s *t* test: *p* = 0.0007, *d* = 0.59, *t* = 3.47). We also found that ApoE4 total dendritic length was significantly reduced (729.6 ± 24.75 µm) when compared with ApoE3 mice (921.4 ± 31.57 µm; Fig. 4*E*; unpaired Student’s *t* test: *p* < 0.0001, *d* = 0.7, *t* = 4.13), which corresponded to an overall 20% decrease. No significant differences were observed in the span of ApoE3 and ApoE4 dendritic trees (Fig. 4*F*; E3, 50.69 ± 1.69 µm; E4, 52.42 ± 1.99 µm; unpaired Student’s *t* test).

We then analyzed the distribution of dendritic trees for adult-born granule cells in the dentate gyrus by performing Sholl analysis (Fig. 4*A*,*B*). Two-way ANOVA revealed a highly significant effect of genotype and the distance from the soma on the number of intersections (*p* < 0.0001, *F*_(1,3834)_ = 112.8; and *p* < 0.0001, *F*_(26,3834)_ = 144.7, respectively), as well as a significant interaction between those two factors (*p* = 0.0013, *F*_(26,3834)_ = 2.048). *Post hoc* tests did not demonstrate differences in the number of proximal dendrites (from 10 to 70 µm) between the different genotypes (Fig. 4*G*; uncorrected Fisher’s LSD *post hoc* tests). However, we observed that ApoE4 adult-born neurons had significantly fewer dendritic branches from 70 to 100 µm from the soma when compared with ApoE3 neurons. For both groups, the dendrites reached the molecular layer, and the number of dendrites peaked at 100 µm for ApoE4 and at 110 µm for ApoE3. In addition, ApoE4 granule neurons demonstrated significantly fewer dendrites than ApoE3 neurons at 100–220 µm from the soma (Fig. 4*G*; uncorrected Fisher’s LSD *post hoc* tests).

### Distribution of adult-born granule cells as a function of their complexity

**Figure 5.**
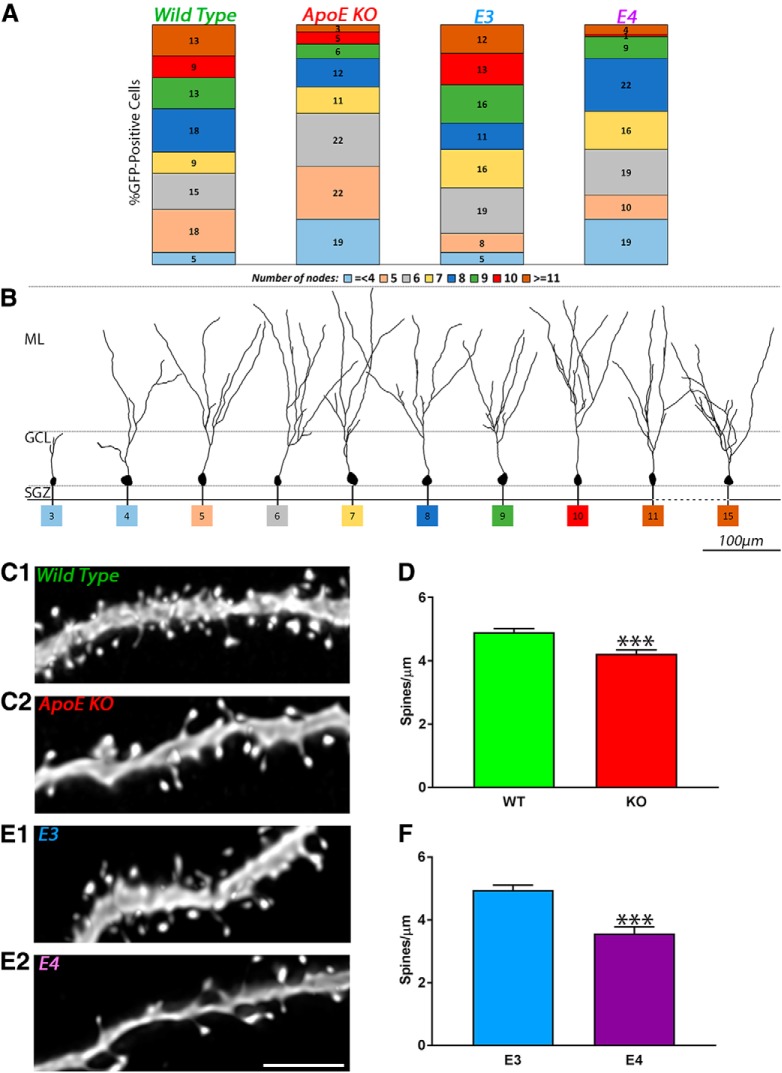
Decrease in the proportion of complex and increase of less complex adult-born granule cells in ApoE-deficient and ApoE4 mice. ***A***, Each population of 3D-reconstructed neurons was divided as a function of the number of nodes (≤4, ≥5, 6…10, 11 nodes or more) and expressed as a percentage of the sample function of the different genotypes: wild type (4 mice, 78 neurons); ApoE-deficient (7 mice, 73 neurons); ApoE3 (6 mice, 75 neurons); and ApoE4 mice (4 mice, 69 neurons). ***B***, Representative pictures of 3D-reconstructed wild-type adult-born granule cells 4 weeks after GFP retroviral infection at different levels of dendritic complexity. ***C1***, ***C2***, ***E1***, ***E2***, Representative pictures of dendritic fragments from wild-type (C1), ApoE-deficient (C2), ApoE3 (E1), and ApoE4 (E2) mature adult-born granule cells. ***D***, ***F***, Spine density quantification in ApoE-deficient (51 fragments from five mice) compared with wild-type (58 fragments from four mice) mice expressed as spines/µm ± SEM, and in ApoE4 (46 fragments from four mice) compared with ApoE3 mice (44 fragments from four mice). Independent *t* test: ****p < 0.0001. Scale bar, ***E2***, 5 µm.

During the acquisition and reconstruction of adult-born granule cells, we observed that the level of complexity of those neurons was heterogeneous. To quantify this observation, we divided the reconstructed neurons as a function of the number of nodes, and we expressed the result as a percentage of the sample (Fig. 5*B*). We demonstrated that 4 weeks after retroviral injections, the average number of nodes of wild-type and ApoE3 adult-born granule cell dendrites is approximately eight (respectively, 7.77 ± 0.29 and 7.87 ± 0.27 µm; Figs. 2*C*, 4*C* ). One observed difference is that ApoE-deficient and ApoE4 mice demonstrate a higher proportion of adult-born granule cells with four nodes (18.84% and 19.18%, respectively; Fig. 5*A*,*B*) compared with wild-type and ApoE3 (respectively, 5.13% and 5.33%). Consistent with this, we observed that ∼50% of wild-type and ApoE3 adult-born granule cell dendritic trees had more than eight nodes compared with 36% for ApoE4 and 26% for ApoE-deficient mice. Therefore, the dendritic trees in both wild-type and ApoE3 newborn neurons were more complex than the ones in ApoE-deficient and ApoE4 adult newborn neurons.

### ApoE regulates spine density

We next investigated the effect of ApoE deficiency and human ApoE isoforms on the density of spines. Because Sholl analysis revealed that the significant difference of dendritic branches was observed at ∼100 µm from the soma, we reconstructed dendritic fragments that were 70–120 µm away from the soma, and analyzed the spine density on randomly selected 10 µm fragments. Adult-born granular neurons in the dentate gyrus of ApoE-deficient mice demonstrated a significantly reduced spine density (4.22 ± 0.12 spines/µm) when compared with wild-type mice (Fig. 5*C*,*D*; 4.90 ± 0.11 spines/µm; unpaired Student’s *t* test: *p* < 0.0001, *d* = 1.13; Fig. 5*C1*,*C2*,*D* ). Similarly, ApoE4 adult-born granule cells also demonstrated a significant decrease in spine density (3.57 ± 0.21 spines/µm) when compared with ApoE3 mice (Fig. 5*E*,*F*; 4.96 ± 0.15 spines/µm; unpaired Student’s *t* test: *p* < 0.0001, *d* = 1.13; Fig. 5*E1*,*E2*,*F* ). Together, those findings demonstrate that the dendritic development of adult-born granule cells in the ApoE-deficient and ApoE4 mouse dentate gyrus is highly impaired.

## Discussion

In this study, we demonstrate that ApoE deficiency leads to a decrease in both the number and activation of type 1 NSPCs in the 10-week-old mouse dentate gyrus. Both ApoE deficiency and ApoE4 lead to less complex mature granule cells in the dentate gyrus as well as reduced spine density at 10 weeks of age. Together, these findings highlight the crucial role of ApoE in adult mouse neurogenesis. We did not perform direct comparisons between wild-type rodent ApoE with human ApoE3 (the predominant and presumed human wild-type allele) and rodent ApoE-deficient and human ApoE4 (the less common and potentially pathogenic). Instead, we aimed to investigate how dentate gyrus adult neurogenesis was affected in the absence of rodent ApoE and between two alleles of human ApoE. The significance of direct comparisons between species is unknown; therefore, we opted not to make them.

Adult hippocampal neurogenesis begins with the activation of quiescent type 1 NSPCs. By performing *in vitro* clonal analysis and *in vivo* unbiased stereology quantification, it has been shown that the total absence of ApoE results in an increased number of active type 1 NSPCs (Yang et al., 2011). The constitutive activation of type 1 NSPCs in ApoE-null mice depletes available type 1 NSPCs over time. Consistent with these findings, we observed a reduction in the number of both total type 1 NSPCs and active type 1 NSPCs in the absence of ApoE. Although different markers were used to determine the number of NSPCs, a similar reduction of NSPCs was observed in another study when older mice were analyzed (Li et al., 2009). Therefore, our present observations are consistent with the requirement of ApoE for the maintenance of quiescent status to prevent the depletion of available NSPCs.

Interestingly, the quantification of BrdU-positive cells and Ki67-expressing cells revealed a difference in ApoE-deficient mice with a significant decrease in Ki67-expressing cells and BrdU-positive cells remaining the same compared with WT mice. It is noteworthy that injections of BrdU were given at 6 weeks of age and observed at 10 weeks of age when Ki67 was detected. A previous study (Yang et al., 2011) suggested that ApoE stimulates neural proliferation in mice that are 1 month of age, while leading to decreased proliferation in mice that are ≥2 months of age, reinforcing the idea that ApoE acts as a negative regulator of neurogenesis and only leads to decreased proliferation at later ages when the progenitor pool is depleted. We hypothesize that we observed no change in BrdU incorporation in mice injected at 6 weeks of age because they were at a time when the effect of ApoE deficiency shifted from stimulating neural proliferation to reducing it, while we found reduced Ki67-expressing cells at 10 weeks of age, a time when ApoE deficiency is correlated with a reduction in proliferation.

The link between the presence of the ApoE4 isoform and the development of Alzheimer’s disease and other cognitive deficits in humans suggests that this isoform impairs normal hippocampal functioning. Despite this correlation, the current study did not find any differences in neuronal specification, hippocampal cell proliferation, proliferation of type 1 NSPCs, or the total number of type 1 NSPCs in the dentate gyrus of 10-week-old mice expressing ApoE4 when compared with those expressing the human wild-type ApoE3. Previous studies present conflicting evidence regarding the role of the ApoE4 isoform in hippocampal neurogenesis. Li et al. (2009) found that the expression of ApoE4 does not affect the proliferation and number of NSPCs, results that are supported by the present study, while increasing overall hippocampal cellular proliferation and inhibiting the maturation of newborn neurons in 6- to 7-month-old mice. However, more recently, it has been reported (Koutseff et al., 2014) that ApoE4 leads to decreased levels of hippocampal cell proliferation in 10- to 12-week-old mice compared with ApoE3 mice, a difference that is attenuated as mice mature. Together, these findings suggest that the effect of ApoE4 on neurogenesis is likely age dependent.

The difference in Ki67-expressing cells between ApoE-deficient mice and ApoE4 mice was also interesting. In the present study ApoE-deficient mice were compared with WT mice to reveal the consequences of absent ApoE, while ApoE4 mice were compared with ApoE3 mice to investigate the effects resulting from different human alleles. Therefore, it is not surprising that we observed a significant decrease of Ki67-expressing cells in ApoE-deficient mice at the chosen time but not in ApoE4 mice. By using another marker, Sox2, to investigate early neural progenitors in ApoE4 and ApoE3 targeted replacement mice, no significant differences were reported (Li et al., 2009). Therefore, while there may be a nominal effect of ApoE4 on neural proliferation, it does not phenocopy the ApoE-deficient state, suggesting more of a hypomorphic role consistent with what we observed in the dendritic and spine analyses.

During normal neurogenesis, adult-born granule cells project their dendrites toward the molecular layer and form synapses on the medial and lateral perforant pathways (Amaral et al., 2007). Morphologic analysis of mature granule cell dendritic arborizations revealed a wild-type phenotype for wild-type and ApoE3-expressing mice and an abnormal phenotype for ApoE-deficient and ApoE4-expressing mice. ApoE4- and ApoE-deficient dentate gyrus adult-born neurons have decreased complexity in their dendritic trees, as well as a decreased total dendritic length. A similar observation was reported in older mice where hippocampal granule cells in ApoE4 mice exhibit less complex dendritic arborizations, which was not observed in ApoE-deficient mice (Li et al., 2009). The present study also highlights that ApoE-deficient and ApoE4 mice have a higher proportion of neurons with less complexity while having a reduced proportion of normal/complex neurons.

The role of cholesterol in neurite outgrowth has been established by culturing retinal ganglion cells with astrocyte-conditioned media (Pfrieger, 2002), and it was subsequently established that astrocyte-secreted cholesterol induced the formation of synapses *in vitro* (Goritz et al., 2005). Because the transportation of cholesterol from astrocytes to neurons is mediated by ApoE-containing lipoproteins, it has been proposed that cholesterol affects neurite formation and synaptogenesis in an ApoE-dependent manner mediated via the low-density lipoprotein receptor (Mamotte et al., 1999). Therefore, it is not surprising that deficits in ApoE could result in impaired neurite formation. Several studies have demonstrated that human ApoE4 inhibits the neurite outgrowth of cultured cortical neurons while ApoE3 promotes it (Nathan et al., 2002). Here we observe that both the deficiency of rodent ApoE and the presence of human ApoE4 resulted in less complexity in newborn neurons, results that are consistent with other findings.

Fewer branches of dendritic trees and lower spine density in newborn neurons in the absence of ApoE and the presence of human ApoE4 were observed when compared with WT and ApoE3 mice. With the narrower angle, fewer dendritic branches, and lower spine density in newborn neurons in the absence of ApoE, neurons born in an ApoE-deficient state are able to receive inputs from both the lateral and medial entorhinal cortex with fewer crossings. However, in the presence of ApoE4, where we observe fewer branches, shorter dendritic length, and lower spine density, the input from the lateral entorhinal cortex may be impaired. Hence, it is not surprising that impairments in performance in hippocampus-dependent behavioral tasks in mice deficient in ApoE or ones carrying the human ApoE4 are observed. Several studies have demonstrated that the presence of human ApoE4 or the absence of rodent ApoE impaired odor memory, Morris water maze task, and contextual fear-conditioning tasks (Masliah et al., 1997; Grootendorst et al., 2001; Peister et al., 2006; Rodriguez et al., 2013; Salomon-Zimri et al., 2015; Peng et al., 2017; East et al., 2018). Further studies are required to determine the electrophysiology of newborn neurons under such genetic backgrounds to see whether they mimic the deficits in behavioral performance.

Dendritic spines are known to be highly variable in size and shape, dynamic, and plastic *in vivo* (Izeddin et al., 2011), and both their morphology and density are abnormal in several neuropsychiatric disorders such as schizophrenia and Alzheimer’s disease (Penzes et al., 2011). The present study revealed a significant decrease in the spine density of both ApoE-deficient and ApoE4 mice, which is consistent with previous work that demonstrated decreased spine density in ApoE-deficient mice at 12 months when compared with wild-type and ApoE3 mice. This decrease was also seen when comparing human AD and age-matched normal control samples carrying one or two copies of the E4 allele compared with samples from individuals without the E4 allele (Ji et al., 2003). Interestingly, the expression of ApoE diminishes when developing into mature neurons from active neural progenitors. The observed deficits in complexity and dendritogenesis in ApoE4 and ApoE-deficient mice might be derived from astrocytic ApoE, as many studies have implicated an astrocytic requirement for proper dendritogenesis (Jain et al., 2013; Perez-Alvarez et al., 2014; Sultan et al., 2015).

Together, our findings reveal a central role of ApoE in dentate gyrus neurogenesis, specifically in the maintenance of the NSPC pool and in the activation of quiescent type 1 cells. Most intriguingly, we observed changes in the morphologic maturation of granule cells as well as their dendritic spine development at a time when they no longer express ApoE, although the proximity of ApoE-expressing astrocytes suggests that, unlike what has been shown with the regulation of NSPC proliferation (Yang et al., 2011), the effect of ApoE on dendritogenesis appears to occur non-cell autonomously, although the mechanism of how this happens remains entirely unknown. We have also shown that human ApoE4 has an effect that is similar to ApoE deficiency in impairing the dendritic maturation of adult-born granule cells, which suggests important consequences at the neuronal network level. We observed these ultrastructural changes in young (6-week-old) mice, far earlier than the onset ApoE4-associated late-onset Alzheimer’s disease. The observations here may represent early lesions that if related to the development of subsequent neurologic disease, may be due to the cumulative effects of these changes over time. Because hippocampal circuitry and neurogenesis play crucial roles in learning and memory, those morphologic aberrances may help to explain, at least in part, the emergence of cognitive decline in humans carrying the E4 allele and provide a link between the E4 allele and hippocampal-related neurologic diseases.

## Synthesis

Reviewing Editor: Julie Andersen, Buck Institute Decisions are customarily a result of the Reviewing Editor and the peer reviewers coming together and discussing their recommendations until a consensus is reached. When revisions are invited, a fact-based synthesis statement explaining their decision and outlining what is needed to prepare a revision will be listed below. The following reviewer(s) agreed to reveal their identity: Jenny Hsieh, Viji Santhakumar.

Both reviewers and myself have now had the opportunity to compare notes on your interesting manuscript submitted to eNeuro exploring the potential link between Apoprotein E (Apo E) status and adult neurogenesis in the murine hippocampus. We felt that the work was an advancement to the field of Alzheimer's disease (AD) research by adding novel information on the potential role of ApoE3 and ApoE4 in adult neurogenesis. There was, however, concern that neither the mechanisms involved nor the functional impact were explored, making the work somewhat limited in scope. Results from the study also raises some important questions in regards to differences between wild type controls, ApoE knockout, and humanized ApoE 3 and 4 knock-in models. Below is a synopsis of specific comments from the group:

Major comments/questions:

1. ApoE-deficient mice demonstrate no change in the production of BrdU-labeled immature neurons, yet there is a drastic decrease in Ki67+ cells in these same animals relative to wildtype (WT) controls. How do the authors explain the maintenance of newly formed neurons in Apo-E deficient mice versus WT in the face of decreased Type 1 cells? Are these Ki67 cells undergoing symmetrical division or asymmetrical division? Is there a compensatory mechanism for immature neuron survival when neural stem cell division is diminished? Is this due to discrepancies between when BrdU was injected (6-weeks old) versus when Ki67 was analyzed (10 weeks old), as perhaps this reduction in neuroproliferation is age-dependent?

2.ApoE4 and ApoE KO exhibit differential changes in neuroproliferation (Ki67), raising the questions of whether these two conditions are comparable; ApoE3 mice display a similar proliferation level compared with ApoE4. The implications of these differences are not explored or discussed.

3. Somatic location of the neurons and if this varied between groups should be addressed.

4.ApoE KO mice exhibit decreased branching and angle, whereas ApoE4 exhibit decreased branching and total dendritic length. The potential mechanisms and /or functional implication should be discussed. It is mentioned in the discussion that ApoE4 and ApoE KO mice exhibit reduced dendritic complexity, however they are not compared to the same control groups.

4. ApoE3 and WT mice would be an important comparison to provide frame of reference for ApoE3 mediated changes in adult-born granule cell morphology and a control for how human ApoE isoforms may affect neurogenesis. This is not included. There are also differences in the distribution of nodes in %GFP-positive cells between WT and ApoE3 in figure 5 (specifically in node categories <4, 5, and 7) that are not discussed.

5. The authors linked the morphological changes observed in ApoE-deficient and ApoE4 mice to hippocampal-dependent spatial memory. Behavioral tests to confirm their hypothesis would greatly increase the overall impact of the manuscript.

6. Figure 5A Y-axis is confusing and should be reconfigured, as the scale is misleading as there are % values in each of the color-coded sections. For instance, the >11 node (brown) section in all groups reaches 100% on the y-axis labeled ‘%GFP-Positive Cells’, yet the percentage values in each of the groups is much lower. It is confusing as to whether the percentage distribution is the value by which each section corresponds to the y-axis or the value labeled within each section (if listed as a percentage, it is assumed to be out of 100).

7. The relevance of the timeline at which morphological changes were examined/identified and the onset of Alzheimer's disease are not discussed.

Minor changes required:

1. In Figure 1 A-D the authors show Nestin, ApoE and GFAP co-localization. High magnification inserts are needed to visualize marker-positive cells.

2. In Figure 2, panels I, J and K are cited in the text before panel A-H. Reorganization of the panels to match the order in the text is needed to avoid misunderstanding.

3. On page 23, ‘pbserved’ need to be changed to ‘observed’.

4. There are extra parentheses in line 5, paragraph 1 in the Introduction in the Hong et al. citation.

5. Spelling error ‘pbserved’ should be changed to ‘observed’ in line 8, paragraph 5 of the Discussion.

## Author Response

Response to Reviewers:

1. ApoE-deficient mice demonstrate no change in the production of BrdU-labeled immature

neurons, yet there is a drastic decrease in Ki67+ cells in these same animals relative to wildtype

(WT) controls. How do the authors explain the maintenance of newly formed neurons in Apo-E

deficient mice versus WT in the face of decreased Type 1 cells? Are these Ki67 cells undergoing

symmetrical division or asymmetrical division? Is there a compensatory mechanism for

immature neuron survival when neural stem cell division is diminished? Is this due to

discrepancies between when BrdU was injected (6-weeks old) versus when Ki67 was analyzed

(10 weeks old), as perhaps this reduction in neuroproliferation is age-dependent?

We appreciate the comment and have added clarification to the manuscript. Other studies have

shown that the effect of ApoE deficiency on neuroproliferation is age-dependent in a way that is

consistent with our findings, presenting a possible explanation for why there are differences

between BrdU+ and Ki67+ cell labeling. Yang et al. (2011), demonstrated that ApoE deficiency

stimulates neuroproliferation in mice that are 1 month of age, while leading to decreased

proliferation in mice that are 2 months of age and older. The basis for this is that it appears ApoE

acts as a negative regulator for Type 1 cell proliferation explaining why proliferation is increased

at early ages and then decreases as the progenitor pool becomes depleted (Yang et, al., 2001).

For our observations, we hypothesize that we found no change in BrdU incorporation in mice

injected at 6-weeks of age because they were injected at a time when the effect of ApoE

deficiency shifts from stimulating neuroproliferation to reducing neuroproliferation, whereas we

found reduced Ki67+ cells at 10-weeks of age, a time when ApoE deficiency is correlated with a

2

reduction in neuroproliferation. We added a paragraph in the Discussion, page 24-25 to address

this issue.

2. ApoE4 and ApoE KO exhibit differential changes in neuroproliferation (Ki67), raising the

questions of whether these two conditions are comparable; ApoE3 mice display a similar

proliferation level compared with ApoE4. The implications of these differences are not explored

or discussed.

This is an interesting point and relates to our response above to point #1. Our results describe a

decrease in NSPC proliferation in the absence of ApoE as compared to WT mice, but no

difference in proliferation when comparing the ApoE3 and ApoE4 targeted-replacement mice,

indicating that ApoE plays a broad role in the maintenance of the NSPC pool, while the ApoE

human isoforms do not differentially affect neuroproliferation. Throughout the manuscript, we

chose to compare ApoE KO and WT mice separately from ApoE3 and ApoE4 targeted

replacement mice, so direct comparisons between ApoE KO and ApoE4 neuroproliferation were

not made in this study.

Other studies have demonstrated that ApoE4 targeted-replacement mice display comparable

levels of NSPC proliferation as ApoE3 targeted-replacement mice, as evidenced by similar

numbers of BrdU/Sox2 double-positive cells (Li et al., 2009). This study, along with our findings,

indicates that the self-renewal of NSPCs in ApoE4 targeted-replacement mice is not altered as

compared to ApoE3 targeted-replacement mice. However, our data indicate that the presence of

ApoE4 does affect dendritic complexity. Taken together, these results suggest that the link

3

between ApoE4 and the development Alzheimer's disease is likely not due to an impairment in

neuroproliferation, but is potentially linked to morphological changes and subsequent aberrant

connections and a decrease in spine density observed in the presence of the ApoE4 isoform. We

added a paragraph in the Discussion (p. 25-26) to address this issue.

3. Somatic location of the neurons and if this varied between groups should be addressed.

It has long been known that newborn neurons in the adult dentate gyrus are restricted to the most

inner aspect of the neuronal layer of the dentate gyrus (Eriksson et al., 1998; Gage et al., 1998).

However, newborn neurons have been observed to migrate into further parts of the granular layer

in injured brains (Ibrahim et al., 2016). In the present work, we only observed newborn neurons

within the innermost aspect of the granular later and we have now explicitly stated this in the

Results (page 18).

4. ApoE KO mice exhibit decreased branching and angle, whereas ApoE4 exhibit decreased

branching and total dendritic length. The potential mechanisms and /or functional implication

should be discussed. It is mentioned in the discussion that ApoE4 and ApoE KO mice exhibit

reduced dendritic complexity, however they are not compared to the same control groups.

We agree that this point needs clarification. The reason we did not perform direct comparisons

between rodent ApoE with human ApoE3 and rodent ApoE-deficient and human ApoE4 is

because that in this study we aimed to investigate how neurogenesis was affected in the absence

4

of rodent ApoE and between two alleles of human ApoE gene and therefore the significance of

direct comparisons between species is unknown and we opted not to make them.

The role of cholesterol in neurite outgrowth has been established by culturing retinal ganglion

cells with astrocyte-conditioned media (Pfrieger et al., 2002) and subsequently it was established

that astrocyte-secreted cholesterol induced the formation of synapses in vitro (Goritz et al., 2005).

Because the transportation of cholesterol from astrocytes to neurons is mediated by ApoE

containing lipoproteins, it has been proposed that cholesterol affects the neurite formation and

synaptogenesis in an ApoE-dependent manner mediated via the low-density lipoprotein receptor

(Mamotte et al., 2008). Therefore, it is not surprising that deficits in ApoE could result in

impairments in neurite formation. Several studies have demonstrated that human ApoE4 inhibits

the neurite outgrowth of cultured cortical neurons while ApoE3 promotes this outgrowth (Nathan

et al., 2002). Our findings that both deficiency of rodent ApoE and the presence of human

ApoE4 resulted in less complexity of newborn neurons, which are consistent with other findings.

Fewer branches of dendritic trees and lower spine density in newborn neurons in the absence of

ApoE and the presence of human ApoE4 were observed when compared with WT and ApoE3

mice. With the narrower angle, fewer dendritic branches, and lower spine density in newborn

neurons in the absence of ApoE, neurons born in an ApoE-deficient state are therefore able to

receive inputs from both the lateral and medial entorhinal cortex with less crossings. However, in

the presence of ApoE4, where we observe fewer branches, shorter dendritic length, and lower

spine density, the input from the lateral entorhinal cortex may be impaired. Hence, it is not

surprising that impairments in performance in hippocampus-dependent behavioral tasks in mice

5

with absence of ApoE or ones carrying the human ApoE4 are observed. Several studies have

demonstrated that the presence of human ApoE4 or absence of rodent ApoE impaired odor

memory, Morris water maze task, and contextual fear conditioning tasks (Masliah et al., 1997;

Grootendorst et al., 2001; Peister et al., 2006; Rodriguez et al., 2013; Salomon-Zimri et al., 2015;

Peng et al., 2017; East et al., 2018). Further studies are required to determine the

electrophysiology of newborn neurons under such genetic backgrounds to see if they reflect

deficits in behavioral performance. We have added a new section in the Discussion highlighting

these points (p. 26 - 27).

5. ApoE3 and WT mice would be an important comparison to provide frame of reference for

ApoE3 mediated changes in adult-born granule cell morphology and a control for how human

ApoE isoforms may affect neurogenesis. This is not included. There are also differences in the

distribution of nodes in %GFP-positive cells between WT and ApoE3 in figure 5 (specifically in

node categories &lt;4, 5, and 7) that are not discussed.

This relates to our response in point #4 above. The reason we did not perform a direct

comparison between rodent ApoE with human ApoE3 is because we aimed to investigate how

the development of adult newborn neurons was affected in the absence of rodent ApoE and

between two alleles of human ApoE gene. The significance of direct comparisons between

mouse and human ApoE is unknown and opted therefore we opted not to make them and instead

emphasize differences between human “WT” ApoE (ApoE3) and human mutant ApoE (ApoE4).

We therefore restricted the comparison to rodent WT versus rodent ApoE and human ApoE3 to

human ApoE4 since ApoE3 and ApoE4 are not normally found in rodents. In Figure 5, we make

6

some quantitative comparisons between the various groups and we have added to the Results

section (p. 22) new analyses that partially quantify some of our observations of differences seen

between ApoE-deficient and ApoE4 mice.

6. The authors linked the morphological changes observed in ApoE-deficient and ApoE4 mice to

hippocampal-dependent spatial memory. Behavioral tests to confirm their hypothesis would

greatly increase the overall impact of the manuscript.

We appreciate this point and although we did not perform our own behavioral tasks, there are

already many studies demonstrating hippocampal-specific behavioral deficits in mice carrying

human ApoE4 or with absence of rodent ApoE. This was addressed in detail above (see

response to #4) and the results of these behavioral tasks were added to the discussion.

7. Figure 5A Y-axis is confusing and should be reconfigured, as the scale is misleading as there

are % values in each of the color-coded sections. For instance, the &gt;11 node (brown) section in

all groups reaches 100% on the y-axis labeled “%GFP-Positive Cells”, yet the percentage values

in each of the groups is much lower. It is confusing as to whether the percentage distribution is

the value by which each section corresponds to the y-axis or the value labeled within each

section (if listed as a percentage, it is assumed to be out of 100).

We appreciate the confusion this figure may have caused and have subsequently revised it by

removing the Y-axis title and horizontal lines to make the chart easier to follow.

7

8. The relevance of the timeline at which morphological changes were examined/identified and

the onset of Alzheimer's disease are not discussed.

Although our findings may help provide insight into the mechanism of human ApoE4 and its

link to the development of late-onset Alzheimer's disease, this was not the point of the study.

Since previous reports described that deficiency of ApoE enhanced proliferation of neural

progenitors at 1-month of age and demonstrated a significant decrease in proliferation at 2

months of age (Mu et al., 2011; Yang et al., 2011) we chose something in between (6 weeks of

age) to determine how young adult neural progenitors respond in the absence of rodent ApoE or

the presence of human ApoE4 and E3. This is a much earlier timepoint than what is observed in

mouse models of Alzheimer's disease and human disease associated with ApoE4. Since the

ultrastructural deficits we observe are relatively modest, it may represent an initial insult where

the cumulative cognitive effects are not noticed until later in life. We have added this to the

Discussion (p. 28-29).

Minor changes required:

1. In Figure 1 A-D the authors show Nestin, ApoE and GFAP co-localization. High

magnification inserts are needed to visualize marker-positive cells.

We have replaced the original picture with one with higher magnification as shown in revised

Figure 1.

8

2. In Figure 2, panels I, J and K are cited in the text before panel A-H. Reorganization of the

panels to match the order in the text is needed to avoid misunderstanding.

We have rearranged the figures to match the description in the Results section as shown in

revised Figure 2 and corresponding figure legends.

3. On page 23, “pbserved” need to be changed to “observed”.

This has been corrected.

4. There are extra parentheses in line 5, paragraph 1 in the Introduction in the Hong et al. citation.

This has been corrected.

5. Spelling error “pbserved” should be changed to “observed” in line 8, paragraph 5 of the

Discussion.

This has been corrected.

9

References

East BS, Fleming G, Peng K, Olofsson JK, Levy E, Mathews PM, Wilson DA (2018) Human Apolipoprotein E Genotype Differentially Affects Olfactory Behavior and Sensory Physiology in Mice. Neuroscience, 380:103–110.

Eriksson PS, Perfilieva E, Bjürk-Eriksson T, Alborn AM, Nordborg C, Peterson DA, Gage FH (1998) Neurogenesis in the adult human hippocampus. Nat Med, 4:1313–1317.

Gage FH, Kempermann G, Palmer TD, Peterson DA, Ray J (1998) Multipotent progenitor cells in the adult dentate gyrus. J Neurobiol, 36:249–266.

Goritz C, Mauch DH, Pfrieger FW (2005) Multiple mechanisms mediate cholesterol-induced synaptogenesis in a CNS neuron. Mol Cell Neurosci, 29:190–201.

Grootendorst J, de Kloet ER, Dalm S, Oitzl MS (2001) Reversal of cognitive deficit of apolipoprotein E knockout mice after repeated exposure to a common environmental experience. Neuroscience, 108:237–247.

Ibrahim AFA, Montojo CA, Haut KM, Karlsgodt KH, Hansen L, Congdon E, Rosser T, Bilder RM, Silva AJ, Bearden CE (2017) Spatial working memory in neurofibromatosis 1: Altered neural activity and functional connectivity. Neuroimage Clin, 15:801–811.

Li G, Bien-Ly N, Andrews-Zwilling Y, Xu Q, Bernardo A, Ring K, Halabisky B, Deng C, Mahley RW, Huang Y (2009) GABAergic interneuron dysfunction impairs hippocampal neurogenesis in adult apolipoprotein E4 knockin mice. Cell Stem Cell 5:634-645.

Mamotte CD, Sturm M, Foo JI, van Bockxmeer FM, Taylor RR (1999) Comparison of the LDLreceptor binding of VLDL and LDL from apoE4 and apoE3 homozygotes. Am J Physiol, 276:E553–7.

Masliah E, Samuel W, Veinbergs I, Mallory M, Mante M, Saitoh T (1997) Neurodegeneration and cognitive impairment in apoE-deficient mice is ameliorated by infusion of recombinant apoE. Brain Res, 751:307–314.

Mu Y, Gage FH (2011) Adult hippocampal neurogenesis and its role in Alzheimer's disease. Mol Neurodegener, 6:85.

Nathan BP, Jiang Y, Wong GK, Shen F, Brewer GJ, Struble RG (2002) Apolipoprotein E4 inhibits, and apolipoprotein E3 promotes neurite outgrowth in cultured adult mouse cortical neurons through the low-density lipoprotein receptor-related protein. Brain Res, 928:96–105.

Peister A, Zeitouni S, Pfankuch T, Reger RL, Prockop DJ, Raber J (2006) Novel object recognition in Apoe(-/-) mice improved by neonatal implantation of wild-type multipotential stromal cells. Exp Neurol, 201:266–269.

10

Peng KY, Mathews PM, Levy E, Wilson DA (2017) Apolipoprotein E4 causes early olfactory network abnormalities and short-term olfactory memory impairments. Neuroscience, 343:364– 371.

Pfrieger FW (2002) Role of glia in synapse development. Curr Opin Neurobiol, 12:486–490.

Rodriguez GA, Burns MP, Weeber EJ, Rebeck GW (2013) Young APOE4 targeted replacement mice exhibit poor spatial learning and memory, with reduced dendritic spine density in the medial entorhinal cortex. Learn Mem, 20:256–266.

Salomon-Zimri S, Liraz O, Michaelson DM (2015) Behavioral testing affects the phenotypic expression of APOE ε3 and APOE ε4 in targeted replacement mice and reduces the differences between them. Alzheimers Dement (Amst), 1:127–135.

Yang C-P, Gilley JA, Zhang G, Kernie SG (2011) ApoE is required for maintenance of the dentate gyrus neural progenitor pool. Development 138:4351-4362.
